# *Salmonella* Strain Specificity Determines Post-typhoid Central Nervous System Complications: Intervention by *Lactiplantibacillus plantarum* at Gut-Brain Axis

**DOI:** 10.3389/fmicb.2020.01568

**Published:** 2020-07-24

**Authors:** Amrita Kaur, Kanwaljit Chopra, Indu Pal Kaur, Praveen Rishi

**Affiliations:** ^1^Department of Microbiology, Panjab University, Chandigarh, India; ^2^University Institute of Pharmaceutical Sciences, Panjab University, Chandigarh, India

**Keywords:** gut-brain axis, *Lactiplantibacillus plantarum*, neurological complications, probiotic, *Salmonella*

## Abstract

Neurological complications occurring due to *Salmonella* infection in some typhoid patients remain a relatively unexplored serious complication. This study firstly aimed to explore whether disseminative ability of *Salmonella* from gut to brain is strain specific or not and on the basis of bacterial load, histopathology, and behavioral changes, it was observed that *Salmonella enterica* serovar Typhimurium NCTC 74 did not cause brain infection in murine model in contrast to *Salmonella* Typhimurium SL1344. Simultaneously, alarming escalation in antimicrobial resistance, making the existing antibiotics treatment inefficacious, prompted us to evaluate other bio-compatible strategies as a potential treatment option. In this context, the role of gut microbiota in influencing behavior, brain neurochemistry, and physiology by modulating key molecules associated with gut-brain axis has captured the interest of the scientific community. Followed by *in vitro* screening of potential probiotic strains for beneficial attributes, efficacy of the selected strain was systematically evaluated at various levels of gut-brain axis against *Salmonella* induced brain infection. Analysis of behavioral (depression, anxiety, and locomotor), neurochemical [gamma amino butyric acid and acetylcholinesterase (AChE)], neuropathological (brain and intestinal histology; bacterial burden), and immunohistochemical studies (tight junction proteins expression) revealed its role in preventing serious manifestations and proving its potential as “psychobiotic.” To the best of our knowledge, this is the first report elaborating strain specificity of *Salmonella* in causing post-typhoidal neurological manifestations and simultaneous use of probiotic in managing the same by influencing the pathophysiology at gut-brain axis.

## Introduction

Occurrence of neurological manifestations in certain patients suffering from typhoid fever has highlighted the ever enhancing complications associated with *Salmonella* infection (Kavaliotis et al., 1994; Szabo et al., 2013). The situation, particularly in infants, presents with a poor prognosis along with relatively high mortality rate and is usually associated with serious side effects that can result in lasting damage (Dewan et al., 2009; Joshi et al., 2011). Incidence of not only typhoidal (Kankananarachchi et al., 2019) but also non-typhoidal serovars (Hanafi et al., 2020) is being increasingly implicated in such manifestations, which has alarmed the scientific community world over (Nadeem et al., 2019). Low index of suspicion, resulting in inability to provide prompt medication by the clinicians, has contributed to adverse outcome in patients (Samal et al., 2009). More importantly, inability of the existing antibiotics in treating such complications along with the emergence and spread of multi-drug resistant *Salmonella*, including colistin resistant (Rule et al., 2019) and extensively drug resistant strains (Khurshid et al., 2019), has worsened the situation, resulting in a serious dearth of effective antimicrobials in the treatment arsenal for this pathogen (Chaudhuri et al., 2018). Therefore, to combat such infections, efforts are being directed by the scientific community toward exploring various biocompatible strategies to which the organisms are not likely to acquire resistance.

Interestingly, various inflammatory conditions targeting the gut have been associated with psychiatric and functional co-morbidity (Bravo et al., 2012; Serra et al., 2019). In this regard, the gut-brain axis or the brain-gut axis has been proposed to be of considerable importance. This complex bi-directional communication occurring between the gut and the brain comprises of the enteric nervous system and the central nervous system (CNS). The communication is aided by various signaling molecules such as neuropeptides and hormones through neuronal, humoral, metabolic, and immune pathways (Saulnier et al., 2013). The importance of this pathway and its role in various physiological functions are also being currently recognized in the infectious etiology of severe acute respiratory syndrome coronavirus-2 (SAR-CoV-2; Bostancıklıoğlu, 2020). Owing to the increasing role of microbes in influencing this bottom-up communication (from gut to brain), the present study has been designed to explore this crosstalk between infection that arises in the gut and its implications in CNS pathology.

The increasing role of gut microbiota in influencing the pathophysiology at the gut-brain axis and its importance in influencing such vital communication systems (resulting in a novel concept of “microbiota-gut-brain axis”) has provided encouraging results, thereby directing future research on this aspect (Collins et al., 2012; Parker et al., 2020). Its crucial role as a key pathway in a wide range of manifestations ranging from inflammatory bowel disease and similar diseases of the gut to metabolic control, ischemic stroke, and various neurodegenerative diseases also encompassing psychology and addictive disorders, has opened wide avenues for targeted interventions in the respective disease states (Liang et al., 2018; Zhao et al., 2018; Grasset and Burcelin, 2019; Jerlhag, 2019; Long-Smith et al., 2020). In this regard, the multi-pronged ability of probiotics in manipulating the intestinal microbiota, resulting in direct impact on the innate immunity, metabolism, and gut physiology, has led to outstanding contributions in treating/preventing a wide spectrum of diseases (Hooper and Gordon, 2001; Sales-Campos et al., 2019). Simultaneously, the role of *Lactobacillus* and *Bifidobacterium* or their metabolites in preventing intestinal infections by inhibiting colonization of pathogenic bacteria such as *Vibrio cholera*, enterotoxigenic *Escherichia coli*, and *Salmonella* has also been studied extensively (Sullivan and Nord, 2002; Rishi et al., 2009, 2011b, 2017). However, recent reports on the ability of probiotics in managing psychological changes occurring due to infections warrant further research (Cryan and Dinan, 2012; Wang et al., 2016). In this regard, probiotics have been named as “psychobiotics” and can serve as a potential treatment intervention in gut-brain axis disorders such as autism and inflammatory bowel disease, along with those mentioned above (Borre et al., 2014; Cheng et al., 2019; Tian et al., 2020).

The present study, thus, elaborates on typhoid induced brain infection using *Salmonella* Typhimurium (known to cause typhoid like disease in murine model) which has been employed as a model for mimicking neurological complications occurring due to typhoid fever in humans (Wickham et al., 2007; Chaudhuri et al., 2018). To the best of our knowledge, this is the first report to explore whether the disseminative ability of *Salmonella* from gut to brain is strain specific or not. Simultaneously, keeping in view the reports implicating molecules of Gram-negative bacteria such as endotoxin in neuropathology associated with Alzheimer’s disease (Zhan et al., 2016), the role of vagus nerve in transmitting abdominal immune information from gut to brain in *S*. Typhimurium infection (Wang et al., 2002), the effect of *Lactiplantibacillus plantarum* (RTA 8) administration on physiological, psychological, and pathological statuses (3Ps) of the diseased host and its role in preventing critical illness by modulating key mediators of the gut-brain axis, was evaluated.

## Materials and Methods

### Bacterial Strains

*Salmonella enterica* serovar Typhimurium NCTC 74, originally obtained from Central Research Institute, Kasauli, India and maintained in our lab since several years, has been used in the present study. For routine experiments, it was maintained on MacConkey agar plates. Another strain of *Salmonella*, namely, *S. enterica* serovar Typhimurium SL1344, kindly provided by Dr. Mrytyunjay Suar, Director, School of Biotechnology, KIIT University, Odisha, was used throughout this study and routinely cultured in Luria-Bertani (LB) broth and on solid LB agar plates containing streptomycin at a concentration of 50 μg/ml. Various strains of *Lactiplantibacillus*, namely, *Lactiplantibacillus pentosus* (9-10), *L. pentosus* (SS), *L. pentosus* (Pc), *L. plantarum* (RTA 8), and *Lactiplantibacillus paraplantarum* (B) (GenBank Accession numbers: KJ802484, KJ802483, KJ802480, KJ802485, and KJ802481, respectively), were kindly provided by Prof. Rupinder Tewari, Department of Microbial Biotechnology, Panjab University, Chandigarh. *Lactococcus lactis* s*ubsp*. *lactis* MTCC 3041 was procured from CSIR-Institute of Microbial Technology, Chandigarh. *L. plantarum* (DM5), *Pediococcus pentosaceus* (CRAG 3), *L. plantarum* NRRL 4496, and *Lactobacillus acidophilus* NRRL 4495 were kindly provided by Dr. Arun Goyal, Professor of Biotechnology, Department of Biosciences and Bioengineering, Indian Institute of Technology, Guwahati, India. All the potential probiotic strains were cultured in de Man, Rogosa and Sharpe (MRS) broth and maintained on MRS plates for routine experiments. Furthermore, all the strains were preserved as 20% glycerol stocks in −80°C deep freezers.

### Animals and Ethics Statement

Five to six weeks old female BALB/c mice weighing 22–26 g, procured from Central Animal House, Panjab University, Chandigarh, were employed in the present study. All animals were adapted to the conditions of animal room for a week prior to initiating any experiments. The animals received standard pellet diet and water *ad libitum*. All the experimental protocols were approved by the Institutional Animal Ethics Committee, Panjab University, Chandigarh, India (Approval No. PU/45/99/CPCSEA/IAEC/2018/225), and guidelines issued by the Committee for the Purpose of Control and Supervision of Experiments on Animals (CPCSEA), Government of India, on animal experimentation, were strictly followed.

### Evaluation of Strain Specificity of *Salmonella* in Causing Brain Infection

To evaluate the disseminative ability of *Salmonella* strains from gut to brain, the animals were divided into four groups containing six mice each and infection studies with *S. enterica* serovar Typhimurium NCTC 74 (routinely employed in our lab) and *S. enterica* serovar Typhimurium SL1344 (reported to cause brain infection in mice; Wickham et al., 2007) were performed. Three doses for each strain were employed and the mice were grouped as follows: (a) **control group**: mice were administered 0.1 ml of phosphate buffered saline (PBS) orally, (b) **infected group I**: mice were orally infected with 0.1 ml of 10^7^ CFU/ml, (c) **infected group II**: mice were orally infected with 0.1 ml of 10^8^ CFU/ml, and (d) **infected group III**: mice were orally infected with 0.1 ml of 10^9^ CFU/ml. Mice were sacrificed at day 7 post infection (systemic spread of the pathogen has been confirmed as per the model standardized in our lab previously; Rishi et al., 2011a; Singh et al., 2013).

Persistence of infection with the selected dose of the selected strain was further checked over a period of 21 days.

#### Evaluation of Behavioral Defects and General Health Score

Mice were observed daily for overt signs of behavior as well as other abnormalities. The clinical symptoms and gross behavioral changes exhibited by mice in all the groups were studied and scored on a general health score (GHS) index of 1–5 on the basis of the following criteria: 5 score = mouse bright-eyed and alert, has a smooth coat with a sheen, responds to stimulus and shows interest in its surroundings; 4 score = fur slightly ruffled, loss of sheen to the coat, mouse remains alert and active; 3 score = fur noticeably ruffled, mouse not as alert and active, less interested in environment outside the cage; 2 score = mouse hunched over and lethargic, little interest shown in environment, fur clumped, signs of hyperventilating when handled; and 1 score = mouse non-reactive to stimulus, fur has a bottle-brush appearance, i.e., standing on end, mouse hunched over, preferring to sleep than react to environment, cold to touch (Gill et al., 2001).

#### Enumeration of Bacterial Load

Mice were sacrificed at day 7 post infection to check for presence of *Salmonella* in the brain as well as other vital organs. Liver, spleen, intestine, and brain of mice in all the groups were rapidly excised and 10%W/V homogenates were prepared in ice cold PBS (0.05 M, pH 7.4) for enumeration of bacterial load in all the organs (Kaur et al., 2017). The homogenates were appropriately diluted and plated on LB agar plates containing streptomycin (50 μg/ml) and kept at 37°C for overnight incubation.

#### Histological Analysis

Brain tissue samples of all the groups were stored in 10% buffered formalin and thereafter processed for histological examination of various parts of the brain by staining with hematoxylin-eosin (H&E) and viewed under the light microscope.

### Screening of Potential Probiotic Strains for Administration in Mouse Model Infected With *Salmonella*

#### Evaluation of *in vitro* Anti-*Salmonella* Activity of Potential Probiotic Strains

All the potential probiotic strains were screened for anti-*Salmonella* activity by performing agar well diffusion assay using tryptone glucose extract (TGE) media. Briefly, the strains were grown for a period of 24 h in MRS broth and the culture was centrifuged for 20 min at 14,050 × *g* to separate the supernatant. The cell free supernatant (CFS) of the strains was further filter sterilized by using 0.22 μm syringe filters prior to its use. 10^7^ CFU/ml of log phase cells of test strain, *S*. Typhimurium SL1344, were seeded into the soft TGE agar (1–1.5% agar concentration) and poured over the glass plates already containing the media (TGE agar-2–2.5% agar concentration). The plates were kept for solidification, after which the wells (7 mm diameter) were punched into them. The lower periphery of the wells were then sealed with 10 μl of 3% agar, following which previously prepared CFS of all the potential probiotic strains were added to the wells. The plates were incubated at 37°C for 24 h. Antimicrobial activity was evaluated by presence or absence of zones of inhibition, the diameter of which was recorded in millimeter (Holder and Boyce, 1994). Sterile MRS broth was used as negative control.

#### Evaluation of Various Functional Attributes of the Potential Probiotic Strains

The strains were screened for their acid tolerance (pH 1, 2, 3, and 4) for a period of 2 h and bile tolerance for a period of 4 h. The samples were taken at regular time intervals and appropriate dilutions were plated on MRS agar and incubated at 37°C for 24 h for enumeration of the surviving population. The strains were further tested for their hydrophobicity by performing Bacterial Adherence to Hydrocarbons (BATH) test using hexadecane and the hydrophobicity index was calculated by using the formula:

$$\%\mathrm{Hydrophobicity}=\left(1-A_{\mathrm{final}}/A_{\mathrm{initial}}\right)\times 100.$$

The test in an indirect assay in the sense that the most hydrophobic strains would have a higher potential to adhere to the intestinal mucosa (Tokatlı et al., 2015).

#### Evaluation of Antimicrobial Substances Responsible for Anti-*Salmonella* Activity of the Potential Probiotic Strains

All the strains were examined for the production of antimicrobial substances such as organic acids, hydrogen peroxide, and bacteriocins. One part of the CFS of all the strains (prepared as described above) was treated with 1N NaOH for neutralization of the acidic component, while another part was treated with catalase to check for production of hydrogen peroxide as the antimicrobial agent. Similarly, to check for the presence of bacteriocins, CFS of all the strains were incubated with enzymes including lysozyme (1 mg/ml), proteinase K (1 mg/ml), pepsin (1 mg/ml), and trypsin (1 mg/ml) for 1 h. CFS after treatment with all the enzymes was then used in agar well diffusion assay against the test pathogen, and the plates were incubated overnight at 37°C. Lysozyme (1 mg/ml), proteinase K (1 mg/ml), pepsin (1 mg/ml), trypsin (1 mg/ml), catalase (1 mg/ml), and 1N NaOH were added in separate wells and served as controls. Untreated CFS served as positive control. The presence/absence of zones of inhibition observed the next day was indicative of the active antimicrobial component responsible for inhibition of the pathogen.

### *In vivo* Studies

Bacterial strains exhibiting the best attributes *in vitro* were chosen to further evaluate their effect on the gut-brain axis. Initially, two strains, namely, *L. pentosus* (9-10) and *L. plantarum* (RTA 8), were administered to mice at a concentration of 10^8^–10^9^ CFU/ml in 0.2 ml of PBS for a period of 7 days before infection and concurrently for a period of 14 days post infection. Negative control (administered with 0.2 ml PBS) and positive infection control (administered with 0.1 ml of *S*. Typhimurium SL1344) group was also put. On the basis of a comparatively better GHS and greater reduction in *Salmonella* bacterial burden in vital organs of mice (on day 7 and day 14 post infection), RTA 8 was chosen for further *in vivo* efficacy testing. Mice were randomly divided into three groups containing 6–8 mice each. **Group I (control)**: mice were administered 0.1 ml of PBS and served as control. **Group II (infected)**: mice in this group were administered 10^8^ CFU/ml of *S. enterica* serovar Typhimurium SL1344 in 0.1 ml of PBS. **Group III (treated)**: mice in this group were administered *L. plantarum* (RTA 8) at a concentration of approx. 10^8^–10^9^ CFU/ml in 0.2 ml of PBS daily for a period of 7 days before infection. Concurrent administration of *L. plantarum* (RTA 8) was carried out for a period of seven more days. Mice in all the groups were sacrificed on day 7 post infection. The vital organs of mice were rapidly excised, weighted, and stored in −80°C until preparation of the homogenates.

#### Evaluation of Systemic *Salmonella* Infection in Terms of General Health Score of Mice and Enumeration of Bacterial Burden in Vital Organs

GHS of mice was recorded daily for the whole duration of the study. The clinical symptoms and gross behavioral changes exhibited by mice in all the groups were studied, and mice were scored on a GHS scale of 1–5 (on same criteria as described before). Simultaneously, the bacterial count in vital organs was assessed after sacrificing mice at day 7 post infection (as described above).

#### Studies Executed at the Gut Axis

##### Evaluation of *Salmonella* Bio-Burden in Intestine

Mice in all the groups were sacrificed at day 7 post infection and homogenates of intestinal tissue were prepared, as described above for enumeration of bio-burden.

##### *In situ* Immunohistochemical Analysis of Tight Junction Proteins Expression in Intestinal Sections of Mice

A section of the intestine was excised from the tissue for estimation of tight junction protein expression. The samples were fixed in phosphate buffered 10% formalin and embedded in paraffin wax, which were cut and mounted on poly L-Lysine (Sigma; Cat No. P8920) coated slides for fixation. The samples were then deparaffinized and sequentially transferred immediately to xylene I, II, and III for 5 min each. The samples were then re-hydrated by sequentially keeping them in 100, 70, and 50% alcohol for 5 min each and then in distilled water. For blocking, the samples were treated with 20 min of bovine serum albumin (BSA). For retrieval of target antigens, the rack of slides containing the samples was immersed in a steel bowl filled with appropriate Tris buffer pH (9.2) and kept in the pressure cooker for 20 min. Two primary antibodies, namely, claudin 5 (ImmunoTag, Cat No. ITT 0953) and occludin (ImmunoTag, Cat No. ITT 7504), diluted to a concentration of 1:50 (concentration was standardized) using 0.1% BSA (diluted with PBS) were used for evaluating the expression of tight junction proteins. Approximately 30 μl of the diluted primary antibody was incubated with the samples in moist chamber for 2 h at room temperature which was flooded with secondary antibody, i.e., fluorescein isothiocyanate (FITC) tagged anti-mouse IgG (Sigma-Aldrich, Cat. No. F0257; diluted to a 1:50 concentration) for detection. Aqueous mounting of the sample was done using 80% phosphate buffer glycerine (PBG). The samples were then stored at 4°C. The slides were viewed under fluorescence microscope (Nikon Eclipse E200, NIS Elements, Version 4.0) to evaluate the change in expression of the specifically targeted antigens.

##### Evaluation of Histological Alterations in Intestine

A part of the intestine tissue samples of all the groups was stored in 10% buffered formalin and processed for histological examination by staining with H&E and thereafter viewed under the light microscope.

#### Studies Executed at the Brain Axis

##### Behavioral Studies to Determine Depression, Anxiety, and Locomotor Function

###### Forced Swim Test

Forced swim test (FST) was performed to assess whether the animals had developed depressive-like behavior or not. The animals were forced to swim for a period of 6 min in a 25 cm × 12 cm × 25 cm apparatus filled with water (temperature: 23 ± 2°C) up to a height of 15 cm. After initial 2 min of acclimatization within the water chamber, the duration for which the mice remained immobile in water during the remaining 4 min was recorded. The animal was considered to be immobile whenever it remained floating passively in the water in a slightly hunched but upright position, with its nose above the water surface. The total immobility time was recorded for all the mice in the groups before initiation of the experiments as well as on day 7 post infection (Sah et al., 2011).

###### Open Field Test

To assess the locomotion and exploratory behavior of mice, open field test (OFT) was performed. The open field box consisted of a square box (600 mm × 600 mm × 200 mm), divided into 16 squares of equal size, made on a black sheet, inside the wooden box. Each animal was placed in the center of the box. The amount of time spent in the center arena, along with the number of rearings and line crossings were recorded using a digital video system for a period of 10 min. After each trial, the test chambers were cleaned with a dry tissue paper, followed by 70% alcohol, and cleaned with the tissue again (Liu et al., 2013).

###### Elevated Zero Maze

Elevated zero-maze (EZM) test was used to examine anxiety-like behavior of mice in all the groups. The maze was divided into four alternating quadrants, two of which had 14 cm high walls (closed arms) and two of which had no walls (open arms). The mice were placed in a closed arm and movement around the maze was video-recorded for a period of 5 min. After each trial, the test chambers were cleaned with a dry tissue paper, followed by 70% alcohol, and cleaned with the tissue again. Number of entries in open arm, time spent in the closed and open arms, and the latency time were evaluated by a blinded observer to assess the behavior (Moon et al., 2015).

##### Estimation of *Salmonella* Bio-Burden in Brain

Mice in all the groups were sacrificed at day 7 post infection and homogenates of brain tissue were prepared, as described above for enumeration of bio-burden.

##### Evaluation of Brain Neurochemistry

Brain neurochemistry of mice in all the groups were studied by evaluating levels of enzyme acetylcholinesterase (AChE) and gamma-aminobutyric acid (GABA) in the brain tissues.

###### Estimation of Brain AChE Activity

Cholinergic dysfunction was evaluated by estimation of AChE activity in the brain tissue. Briefly, brain tissue homogenate prepared in phosphate buffer (pH 8.0) was centrifuged (10 min at 604 × *g*) and, thereafter, 0.05 ml of supernatant was added to the assay mixture containing 3 ml of 0.01 M sodium phosphate buffer (pH 8), 0.10 ml of acetylthiocholine iodide, and 0.10 ml 5,5'-dithiobis-(2-nitro benzoic acid; Ellman’s reagent). The change in absorbance was measured at 412 nm and five consecutive readings were recorded at an interval of 30 s over a period of 2 min. Results were calculated using molar extinction coefficient of chromophore (1.36 × 10^4^ M^−1^ cm^−1^; Tiwari et al., 2009).

###### Estimation of Gamma-Aminobutyric Acid in Brain Tissue of Mice

The brain GABA content was estimated according to the method of Lowe et al. (1958). The brain was rapidly removed and placed in 5 ml of ice-cold trichloroacetic acid (10%W/V), and then homogenized and centrifuged at 14050 × *g* for 10 min at 0°C. From this sample, 0.1 ml of tissue extract was placed in 0.2 ml of 0.14 M ninhydrin solution in 0.5 M carbonate-bicarbonate buffer (pH 9.95). The samples were kept in a water bath at 60°C for 30 min, and then cooled and treated with 5 ml of copper tartrate reagent. After 10 min, fluorescence emission at 377/455 nm was measured in a spectrofluorimeter.

##### *In situ* Immunohistochemical Analysis of Tight Junction Proteins Expression in Cortical Tissue Sections of Brain

A section of the brain cortical tissue from mice in all the groups was excised for estimation of tight junction protein expression (claudin-5 and occludin), as elaborated above.

##### Evaluation of Histological Alterations in Brain Tissue

A part of the brain tissue samples of all the groups was processed for histological examination, as mentioned above.

### Statistical Analysis

All the values have been expressed as mean ± SEM. The behavioral assessments were analyzed using two-way ANOVA followed by Bonferroni post test data analysis using GraphPad Prism software 6.01 (GraphPad Software Inc., La Jolla, CA, USA). Statistical analysis was done by student’s two sample *t*-test, and one-way ANOVA followed by pair wise comparison procedures (Tukey’s test) was used for evaluation of biochemical and bacteriological data. *p* ≤ 0.05 was considered as significant in all the tests.

## Results

### Ability of *Salmonella* to Cause Brain Infection Is Strain Specific

**Figure 1:**
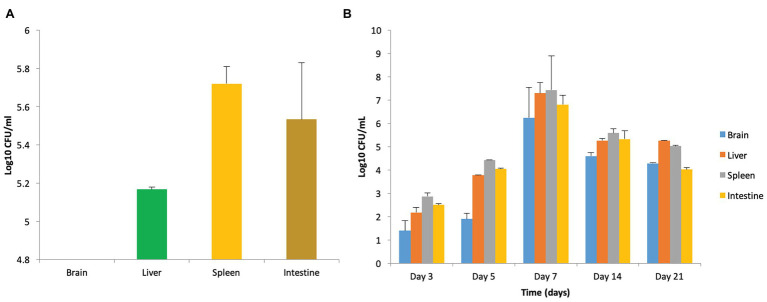
Strain specificity of *Salmonella* in causing murine brain infection. **(A)** Bacterial load in mice when infected with 10^8^ CFU/ml of *Salmonella enterica* serovar Typhimurium NCTC 74 at day 7 post infection. **(B)** Establishment of murine brain infection after oral administration of 10^8^ CFU/ml of *S. enterica* serovar Typhimurium SL1344.

Oral administration of *S. enterica* serovar Typhimurium NCTC 74 resulted in heavy infection in the vital organs such as the liver, spleen, and intestine, validating the systemic spread of the pathogen. However, it did not cause infection of the brain as it was found to be sterile (Figure 1A) even at higher doses of 10^8^ and 10^9^ CFU/ml. The infected mice were observed to be lethargic and demonstrated reduced eating/drinking but no balance defect was seen. The observations indicate that systemic dissemination of the pathogen may not necessarily cause CNS infection. Therefore, this strain was not employed for further studies.

On the contrary, oral infection with *S. enterica* serovar Typhimurium SL1344 resulted in murine brain infection. Out of three doses used for standardization of the infection model, 10^8^ CFU/ml was chosen as the infectious dose on the basis of behavioral defects, bacterial load, histopathological analysis, and mortality rate, as explained below.

#### Behavioral Defects and GHS of Mice Infected With *Salmonella* Typhimurium SL1344

Mice infected with 10^7^ CFU/ml demonstrated reduced eating/drinking. Since brain infection was observed infrequently in this group, therefore the same was not chosen for further studies. Mice administered with 10^9^ CFU/ml were found to be extremely sick with greatly reduced locomotor activity and a significant reduction in eating/drinking. Due to high morbidity, they were also not chosen for further studies. Mice infected with 10^8^ CFU/ml also demonstrated reduced movement and a decrease in the intake of food and water but, in contrast to mice infected with 10^7^ and 10^9^ CFU/ml 20% of them were found to exhibit a balance defect, wherein exaggerated leaning of mice on one side was observed. On studying the GHS of mice, a score of 1–3 was assigned to all the animals in this infected group.

#### Bacterial Load in Vital Organs of Mice Infected With *Salmonella*

Enumeration of bacterial burden in the brain tissues of mice in all the groups revealed that incidence of brain infection was more frequently observed in mice receiving 10^8^ CFU/ml in comparison to the group receiving 10^7^ CFU/ml. Though, mice infected with 10^9^ CFU/ml also demonstrated brain infection but were not chosen due to high mortality. Furthermore, persistence of infection in mice infected with 10^8^ CFU/ml was confirmed on the basis of enumeration of bacterial bio-burden in the brain, liver, spleen, and intestine over a period of 21 days (Figure 1B).

#### Histopathological Analysis of Brain Tissue Confirming Encephalitis and Meningitis

**Figure 2:**
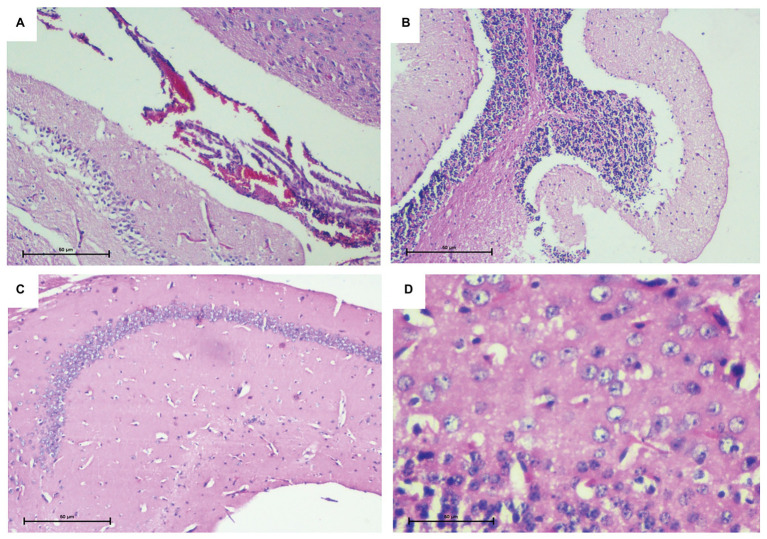
Histopathological analysis of brain tissue confirming murine brain infection. **(A)** Piamater and cortex showing heavy inflammation. **(B)** Spongiosis in cerebellum with infiltration of inflammatory cells. **(C)** Uninfected mouse brain tissue depicting a normal hippocampus devoid of any inflammation. **(D)** Uninfected mouse brain tissue depicting normal cortex and presence of astrocytes.

Murine brain infection was further confirmed on the basis of histological analysis of the brain tissue sections. All parts of the brain including the cortex, cerebellum, meninges, and the ventricle wall were found to be heavily infiltrated. In contrast to control group, inflammation in piamater, cortex, and cerebellum of infected mice confirmed brain infection in the infected group (Figure 2).

#### Mortality Rate of Mice Infected With *Salmonella*

The group receiving 10^9^ CFU/ml of *Salmonella* was associated with a high mortality rate of around 40% and therefore, was not employed for further studies. Mice in this group succumbed to infection as early as day 2 post infection. On the contrary, mice receiving 10^8^ CFU/ml presented with a 20% mortality rate.

### Selection of Potential Probiotic Strain for Administration in Mouse Model Infected With *Salmonella*

#### Anti-*Salmonella* Activity of Various Potential Probiotic Strains

**Figure 3:**
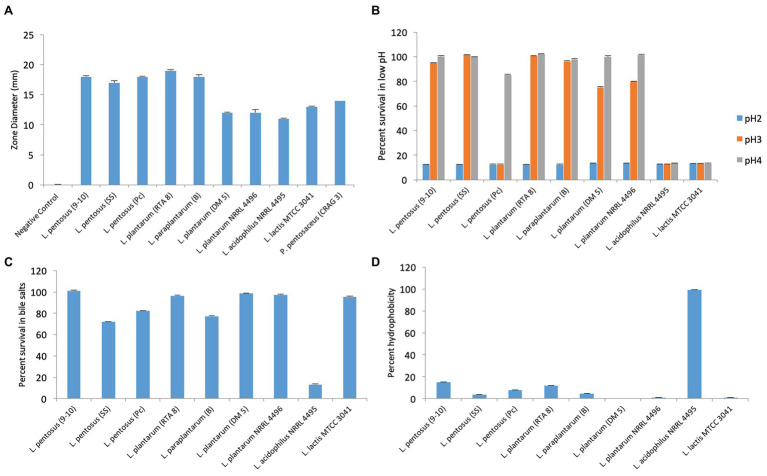
Evaluation of various attributes of probiotic candidates used in the study. **(A)** Evaluation of anti-*Salmonella* activity exhibited by cell free supernatants of various bacterial strains. **(B)** Percent survival of bacterial strains at various pH values. **(C)** Percent survival of bacterial strains at 0.3% bile concentration. **(D)** Percent hydrophobicity of various bacterial strains by bacterial adherence to hydrocarbons (BATH) test.

Among the various strains which were screened for secretion of anti-*Salmonella* activity, *L. pentosus* (9-10), *L. plantarum* (RTA 8), *L. pentosus* (Pc), and *L. paraplantarum* (B) gave the maximum zone of inhibition, thereby indicating most potent anti-*Salmonella* antibacterial activity followed by *L. pentosus* (SS). *L. acidophilus* NRRL 4495 gave the least activity (Figure 3A).

#### Functional Attributes of the Potential Probiotic Strains

The strain exhibiting the best attributes *in vitro* was to be chosen for further *in vivo* studies. Therefore, the strains were assessed for their ability to tolerate acidic conditions (pH 2, 3, and 4), bile salt concentration of 0.3% and ability to adhere to hydrocarbons. The strains could not survive at pH 2 but at pH 3, the best survivability was exhibited by SS followed by RTA 8, B, and 9-10 in the same order. Pc, *L. acidophilus* NRRL 4495, and *L. plantarum* NRRL 4496 could not survive at pH 3 and pH 4 (Figure 3B). The strains, RTA 8, *L. lactis* 3041, *L. plantarum* NRRL 4496, *L. plantarum* DM5, and 9-10 exhibited a survivability of 95–100% in bile salts (Figure 3C). Unlike other attributes, *L. acidophilus* NRRL 4495 gave the best hydrophobicity index of the order of 99% and was found to be between 10–15% in case of RTA 8 and 9-10. The rest of the strains showed an even lower hydrophobicity index (Figure 3D). Taking into consideration the overall properties, two strains, namely, RTA 8 and 9-10, were found to exhibit the best attributes and the most potent anti-*Salmonella* activity *in vitro* and therefore were chosen for *in vivo* studies.

#### Acidic Component in CFS of Potential Probiotic Strains Contributes to Anti-*Salmonella* Activity

Except for the wells that contained the CFS which had been neutralized with 1N NaOH, every other well containing the CFS (after treatment with various proteolytic enzymes) was found to exhibit the same size of the zone of inhibition (with a slight difference of 1–2 mm), indicating that presence of acidic components in the CFS was majorly responsible for the antibacterial activity of the strains against *Salmonella* (Table 1).

**Table 1:** Evaluation of active antimicrobial component in the cell free supernatant of various potential probiotic strains after treatment with various agents.

Potential probiotic strains	Untreated CFS controls	NaOH (1 N)	Catalase (1 mg/ml)	Lysozyme (1 mg/ml)	Proteinase K (1 mg/ml)	Trypsin (1 mg/ml)	Pepsin (1 mg/ml)
*L. pentosus* (9-10)	+	−	+	+	+	+	+
*L. pentosus* (SS)	+	−	+	+	+	+	+
*L. pentosus* (Pc)	+	−	+	+	+	+	+
*L. plantarum* (RTA 8)	+	−	+	+	+	+	+
*L. paraplantarum* (B)	+	−	+	+	+	+	+
*L. plantarum* (DM5)	+	−	+	+	+	+	+
*L. plantarum* NRRL 4496	+	−	+	+	+	+	+
*L. acidophilus* NRRL 4495	+	−	+	+	+	+	+
*L. lactis* MTCC 3041	+	−	+	+	+	+	+

### *In vivo* Efficacy of *L. plantarum* (RTA 8) Against *Salmonella* Induced Murine Brain Infection

**Figure 4:**
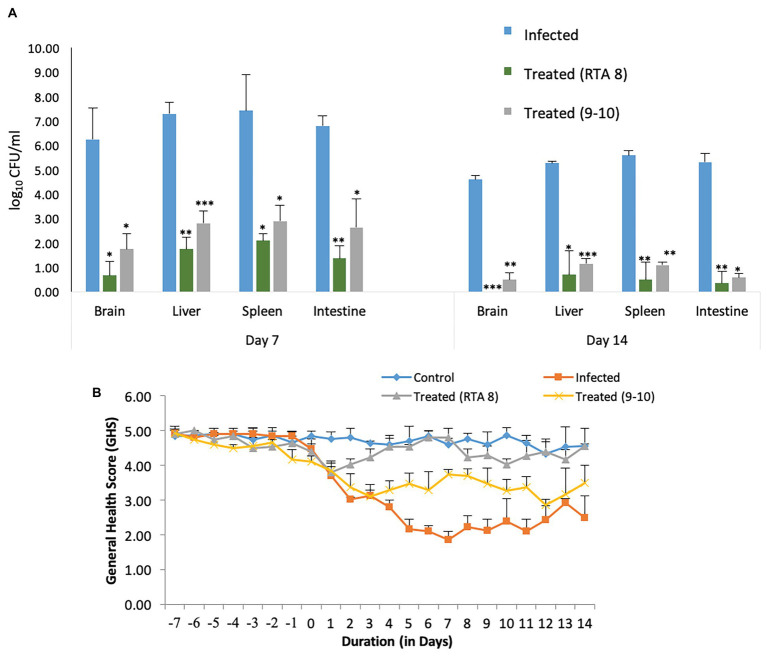
Comparative efficacy of *Lactiplantibacillus plantarum* (RTA 8) and *Lactiplantibacillus pentosus* (9-10) in terms of reduction in bacterial burden and general health score (GHS). **(A)** Pre-administration of *L. plantarum* (RTA 8) 7 days before infection and concurrently for a period of 14 depicts higher significant reduction in the bio-burden of mice in various organs than the group treated with *L. pentosus* (9-10). ^*^p < 0.05, ^**^p < 0.01, and ^***^p < 0.001 infected vs treated. **(B)** Better GHS was observed in mice treated with *L. plantarum* (RTA 8) than those treated with *L. pentosus* (9-10).

Even though both the strains, RTA 8, and 9-10 were found to significantly reduce the bacterial burden, but RTA 8 demonstrated better efficacy in terms of reduction in bio-burden and improvement in GHS when administered to mice infected with *Salmonella* (Figure 4). Therefore, RTA 8 was chosen for further *in vivo* experiments.

#### Efficacy of *L. plantarum* (RTA 8) Administration in Combating Systemic *Salmonella* Infection in Terms of GHS and Tissue Bio-Burden

**Figure 5:**
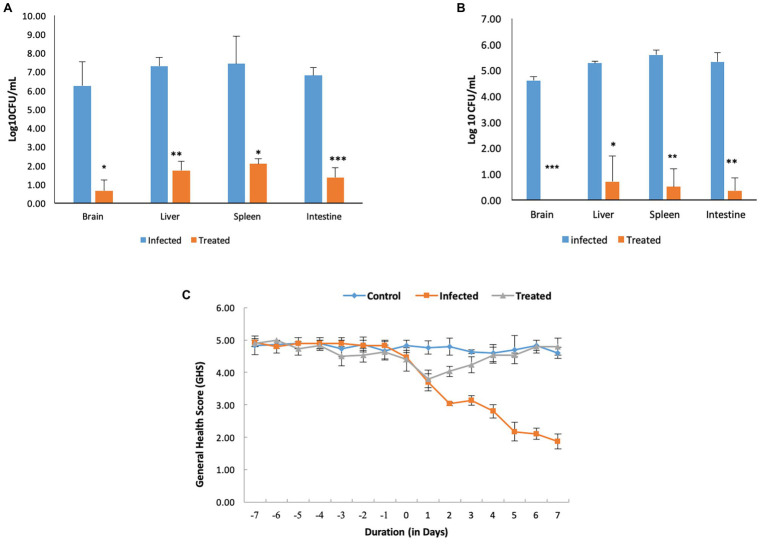
Pre-treatment and concurrent administration of *L. plantarum* (RTA 8) decreases the severity of infection. **(A)** Pre-administration of *L. plantarum* (RTA 8) 7 days before infection and concurrently for a period of 7 days more after infection significantly reduces the bio-burden in vital organs. **(B)** *L. plantarum* (RTA 8) administration for a period of 14 days after infection results in clearance of infection from the brain along with resulting in a more significant reduction in bacterial burden in other vital organs. ^*^p < 0.05, ^**^p < 0.01, and ^***^p < 0.001 by ANOVA. **(C)** Mice in all the groups were observed daily for the whole duration of the experiment and graded on a scale of 1–5, as described in Materials and Methods section.

Most of the mice in the infected group could be included in the low GHS of 1–3. Along with the attributes assigned to each score, mice in this group were found to exhibit reduced eating and drinking with redness around eyes. The situation was accompanied by weight loss. On the contrary, most of the mice in the treated group were assigned with a score of 5 and 4. The mice in this group were found to be alert and active and their weight was found to be almost constant with some showing an increase (Figure 5C). No morbidity or balance defect was observed in animals receiving *L. plantarum* (RTA 8) and was also associated with 100% survivability in contrast to the infected group, which demonstrated survivability of 80% and balance defect in 20% of the mice in the group. Simultaneously, *L. plantarum* (RTA 8) administration resulted in a significant bacterial burden reduction in the liver and spleen tissues of infected mice, as demonstrated by a 4.18 (*p* < 0.01) and 3.54 (*p* < 0.05) fold reduction, respectively, after 14 days of administration (Figure 5A). The bio-burden was found to be further reduced after seven more days of *L. plantarum* (RTA 8) administration with a further 7.54-fold (*p* < 0.05) and 11.17-fold (*p* < 0.01) reduction in the liver and spleen, respectively, thereby proving the efficacy of *L. plantarum* (RTA 8) in combating *Salmonella* infection (Figure 5B).

#### Effect of *L. plantarum* (RTA 8) in Ameliorating *Salmonella* Induced Disturbances at the Gut Axis

##### Effect of *L. plantarum* (RTA 8) Administration on *Salmonella* Bio-Burden in Intestine

Administration of RTA 8 for a period of 7 days prior to infection and continuously thereafter for a period of seven more days resulted in a significant reduction in bacterial load in the intestine of the infected mice. On day 7 post infection, 4.94-fold (*p* < 0.001) log unit reduction was observed in the intestine of all mice in the treatment group (Figure 5A). Concurrent administration of the strain for a period of seven more days resulted in further 15.23-fold (*p* < 0.01) reduction in *Salmonella* bio-burden from the infected tissue (Figure 5B).

##### *In situ* Immunohistochemical Analysis of Effect of *L. plantarum* (RTA 8) Administration on Tight Junction Proteins Expression in Intestinal Tissue Sections of *Salmonella* Infected Mice

**Figure 6:**
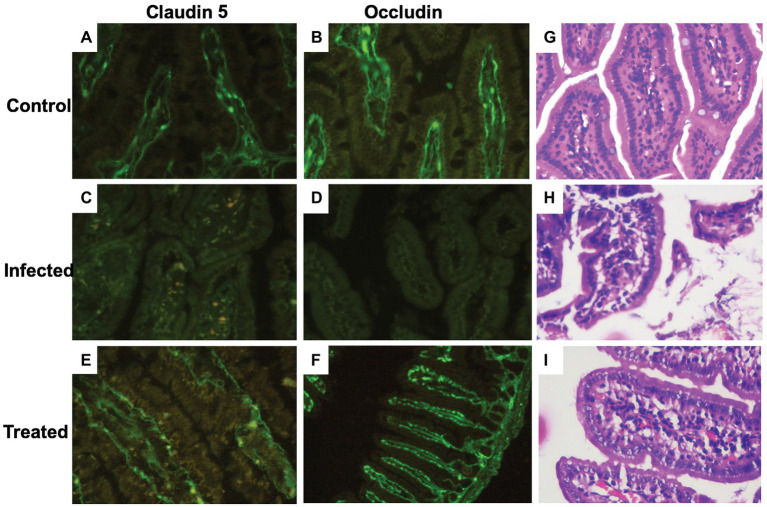
Immunohistochemical and histological analysis of intestinal tissue sections. **(A,B)** Photomicrographs reveal normal expression of the tight junction proteins (claudin-5 and occludin) in the control group. **(C,D)** A highly significant reduction in tight junction proteins can be visualized in the infected group. **(E,F)** Tissue sections of *L. plantarum* (RTA 8) administered mice revealed normal and intact as well as continuous expression of the tight junction proteins. **(G)** (×40, scale bar 50 μm) Microscopic appearance of the intestinal section after hematoxylin-eosin (H&E) staining depicting normal tissue morphology. **(H)** (×40, scale bar 50 μm) The villi of the intestine depict decreased density of goblet cells and the density of lymphocytes in the lamina propria are heavier than normal depicting ileitis highlighting the damage inflicted by *Salmonella* in the infected group. **(I)** (×40, scale bar 50 μm) Intestinal sections of the *L. plantarum* (RTA 8) treated group revealing presence of normal goblet cells along with reduction in the lymphocytes validating the efficacy of *L. plantarum* (RTA 8).

Tissue sections of the intestine in the control group depicted a uniform distribution of the tight junction proteins, claudin-5 and occludin, along with an intense staining intensity confirming normal expression of the selected proteins (Figures 6A,B). On the contrary, photomicrographs of the infected tissue sections depicted a significant reduction in the expression of these proteins. Crypt shortening and destruction of the normal villi morphology along with presence of debris in the crypts were also seen in the intestinal sections of the mice infected with *S. enterica* serovar Typhimurium SL1344 (Figures 6C,D). *L. plantarum* (RTA 8) administration not only resulted in uniform expression of both the proteins but also depicted increased staining intensity in the intestinal tissue sections, thereby indicating an increase in the expression of these proteins. The intestinal crypts depicted normal tissue morphology and the surface proteins were found to be fully restored (Figures 6E,F).

##### Effect of *L. plantarum* (RTA 8) Administration on *Salmonella* Induced Histological Changes in Intestine

In contrast to the control group (Figure 6G), histological analysis of the intestinal sections in the infected group revealed severe damage to the crypts. The villi were discontinuous and surface of the villi was found to be degenerated with heavy influx of inflammatory cells in the mucosal tissue (Figure 6H). In contrast to this, the histological analysis of mice administered with *L. plantarum* (RTA 8) revealed intact mucosal layer with continuous villi. There was no debris or presence of any vacuoles validating the efficacy of *L. plantarum* (RTA 8) administration in rectifying the damage induced by *Salmonella* (Figure 6I).

#### Effect of *L. plantarum* (RTA 8) in Ameliorating *Salmonella* Induced Disturbances at the Brain Axis

##### Effect of *L. plantarum* (RTA 8) in Reversing Behavioral Abnormalities in *Salmonella* Infected Mice

###### Effect of *L. plantarum* (RTA 8) in Reducing Depressive-Like Behavior.

**Figure 7:**
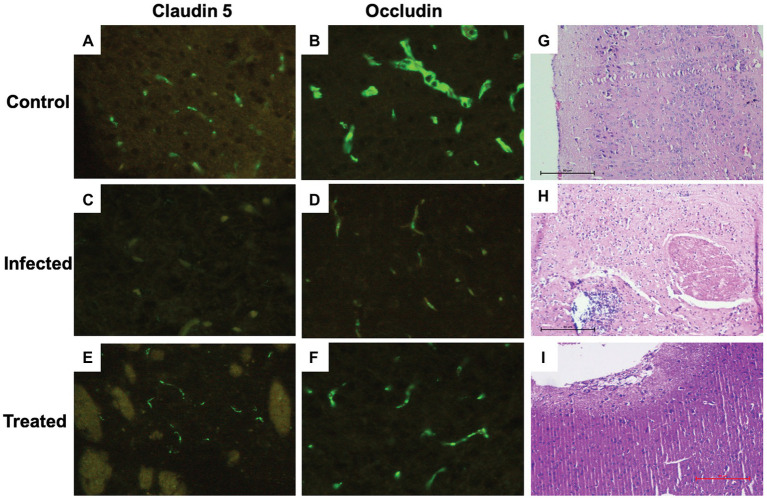
Effect of *L. plantarum* (RTA 8) administration on behavior of mice. Effect on exploratory and locomotor aspect in open field test on the basis of **(A)** no. of line crossings. ^***^p < 0.001 control vs. infected. ^#^*p* < 0.05 control vs. treated. ^$$$^*p* < 0.001 infected vs. treated. **(B)** No. of rearings. ^***^p < 0.001 control vs. infected. ^#^*p* < 0.05 control vs. treated. ^$$$^*p* < 0.001 infected vs. treated. **(C)** Time spent in inner squares. ^**^p < 0.01 control vs. infected. **(D)** Significant decrease in depressive-like behavior in mice was evidenced by a significant decrease in the immobility time in the forced swim test upon *L. plantarum* (RTA 8) administration. ^***^p < 0.001 control vs. infected. ^$$$^*p* < 0.001 infected vs. treated. Reduction in anxiety-like behavior was evidenced in the elevated zero maze test on the basis of **(E)** No. of rearings. ^***^p < 0.001 control vs. infected. ^$$^*p* < 0.01 infected vs. treated. **(F)** No. of head dips. ^**^p < 0.01 control vs. infected. ^$^*p* < 0.05 infected vs. treated. **(G)** No. of entries in open arm. ^##^*p* < 0.01 control vs. treated. ^$$^*p* < 0.01 infected vs. treated. **(H)** Latency time. ^***^p < 0.001 control vs. infected. ^$$$^*p* < 0.001 infected vs. treated and **(I)** time spent in open arm. ^***^p < 0.001 control vs. infected. ^$$$^*p* < 0.001 infected vs. treated.

Mice in the infected group were found to exhibit a significant depressive-like behavior, as evidenced by an increase in the immobility time in the FST (*p* < 0.001). The same was reversed in mice administered with *L. plantarum* (RTA 8). In fact, the mice in this group were found to be more active than the control group (Figure 7D).

###### Effect of *L. plantarum* (RTA 8) in Increasing Exploratory Behavior.

In OFT, reduced locomotor activity/exploratory behavior was evidenced by a highly significant decrease in the number of rearings (*p* < 0.001; Figure 7B), line crossings (*p* < 0.001; Figure 7A), and time spent in inner squares (*p* < 0.01; Figure 7C) in the infected group as compared to the control group. A very significant increase in the number of rearings and line crossings (*p* < 0.001) was observed in the treatment group in comparison to the infection group after *L. plantarum* (RTA 8) administration. However, there was no significant difference in the time spent in the inner squares in both the groups.

###### Effect of *L. plantarum* (RTA 8) in Reducing Anxiety-Like Behavior.

Mice in the infected group demonstrated a highly anxiety-like behavior with a significant decrease in the number of head dips (*p* < 0.01; Figure 7F) and rearings (*p* < 0.001; Figure 7E), simultaneously demonstrating an increase in the latency time (*p* < 0.001; Figure 7H) in the EZM test. There was no significant difference in the number of entries in open arm (Figure 7G), although a very significant decrease in the time spent in the open arm (*p* < 0.001; Figure 7I) was recorded in mice infected with *Salmonella*. Administration of *L. plantarum* (RTA 8) resulted in a very significant decrease in the anxiety-like behavior experienced by mice in all the groups. This anxiolytic effect was demonstrated by a significant increase (*p* < 0.01) in the number of entries in the open arm by mice in the treated group in comparison to those in the infected group. In fact, mice in the treatment group demonstrated even lower level of anxiety than the control group (*p* < 0.01). A significant increase in the number of head dips (*p* < 0.05), rearings (*p* < 0.01), and time spent in open arm (*p* < 0.001) with simultaneous decrease in the latency time (*p* < 0.001) in the treated group in comparison to the infected group, confirmed the ability of *L. plantarum* (RTA 8) in ameliorating the level of anxiety experienced by the animals.

##### Effect of *L. plantarum* (RTA 8) Administration on *Salmonella* Bio-Burden in Brain

Administration of RTA 8 for a period of 7 days prior to infection and continuously thereafter for a period of seven more days resulted in a significant reduction in bacterial load in the vital organs of mice including the brain. On day 7 post infection, a 9.37 (*p* < 0.05) log unit reduction was observed in the brain tissue of mice in the treatment group (Figure 5A). Further, administration of *L. plantarum* (RTA 8) for a period of seven more days resulted in clearance of bacteria (*p* < 0.001) from the brain tissues of animals in the treatment group, thereby validating the efficacy of the same (Figure 5B).

##### Effect of *L. plantarum* (RTA 8) Administration on *Salmonella* Induced Changes in Brain Neurochemistry

Analysis of levels of AChE and GABA in the brain tissues was performed after administration of *L. plantarum* (RTA 8).

###### Effect of *L. plantarum* (RTA 8) Administration on Level of Brain Acetylcholinesterase.

**Figure 8:**
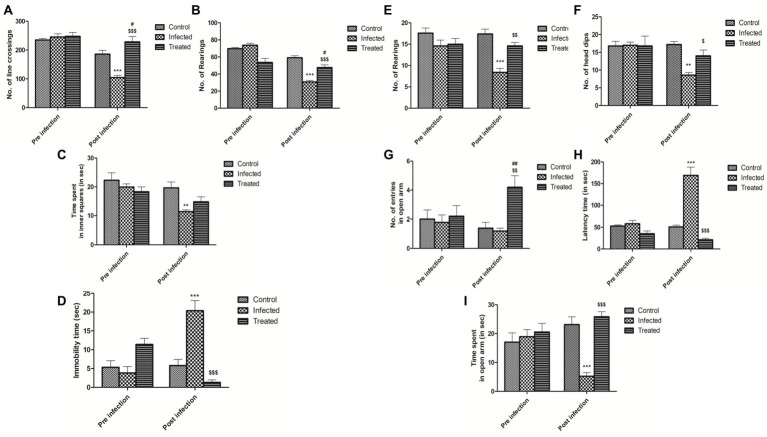
Effect of *L. plantarum* (RTA 8) administration on brain neurochemistry. **(A)** Level of AChE in the murine brain tissue. A significant increase in the level of the enzyme was observed in the infected group which was normalized after administration of *L. plantarum* (RTA 8). Control vs. infected ^**^p < 0.01. **(B)** Levels of GABA in the murine brain tissue. A significant decrease in the level of GABA was observed in the infected group which was normalized after *L. plantarum* (RTA 8) administration. Control vs. infected ^*^p < 0.05.

A significant increase (*p* < 0.05) in the level of AChE was observed in the brain of mice infected with *S*. Typhimurium SL1344. The same was reversed in mice treated with *L. plantarum* (RTA 8) and the levels were found to be similar to those in the control group (Figure 8A).

###### Effect of *L. plantarum* (RTA 8) Administration on Level of Gamma-Aminobutyric Acid.

In contrast to the control tissue, a significant reduction (*p* < 0.05) in the *Salmonella* infected mice was observed in levels of GABA. Administration of *L. plantarum* (RTA 8) resulted in reversal of the deficit as the levels were found to be increased in comparison to the control group (Figure 8B).

##### *In situ* Immunohistochemical Analysis of Effect of *L. plantarum* (RTA 8) Administration on Tight Junction Proteins Expression in Brain Sections of *Salmonella* Infected Mice

**Figure 9:**
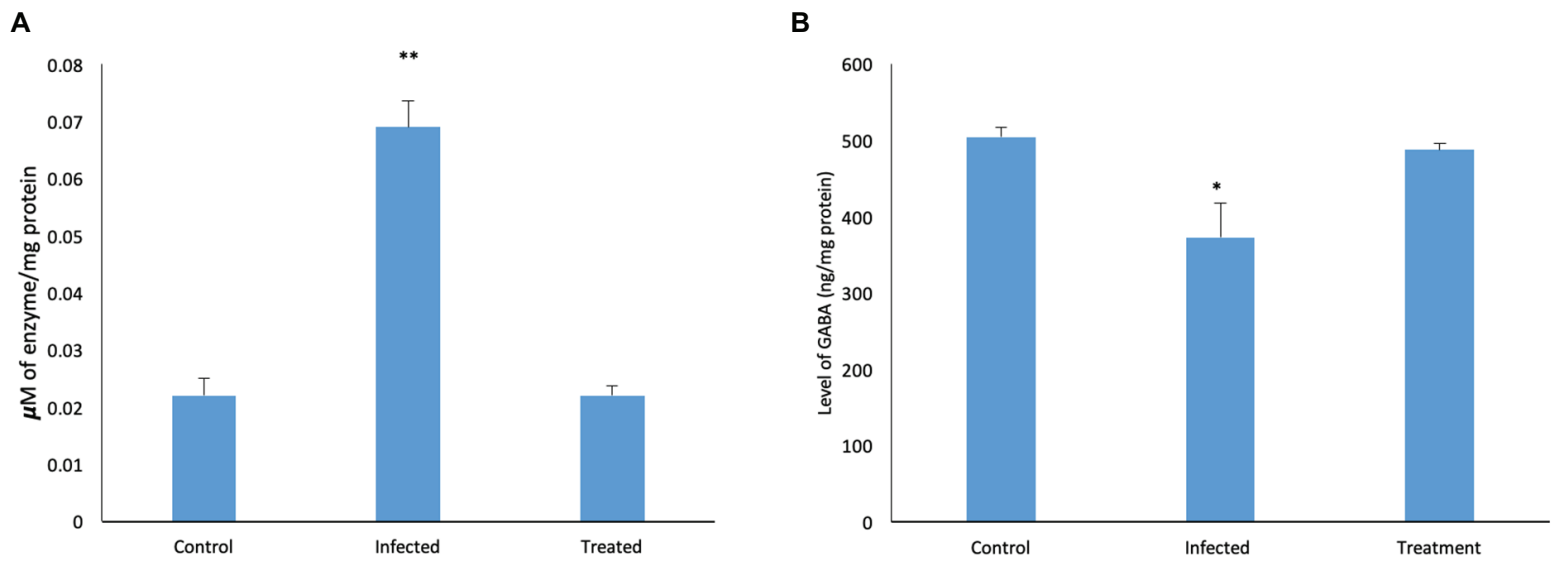
Immunohistochemical and histological analysis of brain tissue sections. **(A,B)** Brain tissue sections of the control group depicting uniform expression of tight junction proteins-claudin-5 and occludin. **(C,D)** A highly significant reduction in the expression of tight junction proteins can be visualized in the *Salmonella* infected group. **(E,F)** Increased expression of tight junction protein expression can be observed in the group treated with *L. plantarum* (RTA 8). **(G)** (×100, scale bar 50 μm) Cortical tissue section of mice in the control group depicting normal histo-architecture. **(H)** (×100, scale bar 50 μm) The tissue sections of the infected group revealed heavy inflammation in all parts of the brain and in certain areas, abscess formation can also be seen along with loss of surface piameter suggesting encephalitis. **(I)** (×100, scale bar 50 μm) Mice administered with *L. plantarum* (RTA-8) depicted normal brain histoarchitecture except for a small supra-cerebral focus of inflammation.

In contrast to the control group (Figures 9A,B), photomicrographs of the infected tissue sections depicted a significant reduction in the expression of these proteins. A reduction in staining intensity was indicative of a substantial loss of proteins at the surface, and complete loss of the junctional proteins at some areas was evidenced by complete loss of staining (Figures 9C,D). Difference in expression was more pronounced in case of claudin-5 in comparison to occludin (Figure 9). *L. plantarum* (RTA 8) administration not only resulted in uniform expression of both the proteins but also depicted increased staining intensity, thereby indicating an increase in the expression of these proteins (Figures 9E,F).

##### Effect of *L. plantarum* (RTA 8) Administration on *Salmonella* Induced Histological Changes in Brain

In contrast to the mice brain tissue sections in the control group, which depicted normal hippocampus and cortex with presence of triangular shaped neuronal cells and astrocytes along with microglial cells and the oligodendrocytes (Figure 9G), the tissue sections of mice in the infected group showed signs of heavy inflammation in the brain. The cortex showed visible signs of necrosis, spongiosis, and presence of inflammatory cells (Figure 9H). Unlike the infected tissue, mice in the treated group showed slight inflammation in the piamater with a small focus of inflammation with increased vascularity on the surface of the parietal cortex. The remaining part of the brain was found to depict normal morphology sans inflammation (Figure 9I).

## Discussion

The human body has always been an intensely intriguing subject and equally fascinating intricate communications occurring within it. Recent discoveries in germ-free animal models have highlighted the role of gut microbiome in influencing behaviors, brain neurochemistry, and physiology (Cryan and Dinan, 2012). Simultaneously, the occurrence of Gram-negative bacterial molecules such as LPS and K99 pilli protein from *E. coli* in the brain tissue of humans presenting with Alzheimer’s disease (Zhan et al., 2016) presents an important link for exploring the possibility of prolonged inflammation or changes in the gut-microbiome caused due to such infections, eventually manifesting into neurological complications. In view of these indications, post typhoidal complications resulting in CNS pathology were studied.

Interestingly, in the present study, *in vivo* evaluation of two different strains revealed that in contrast to *S. enterica* serovar Typhimurium SL1344 (Wickham et al., 2007), administration of *S. enterica* serovar Typhimurium NCTC 74 (which has previously been employed in our lab for various systemic typhoid related studies) did not cause brain infection in murine model. Administration of wild *Salmonella* strains by intraperitoneal, oral, and intranasal route has been reported to reach the brain tissue (Bollen et al., 2008) but the inability of the wild strain *S*. Typhimurium NCTC 74 in causing brain infection unlike *S*. Typhimurium SL1344 might be due to difference in individual virulence of these strains. The observation suggests the possibility of certain strain specific molecular mechanisms which might result in such serious manifestations, as seen in the latter. The mechanism behind spread of the pathogen from the gut to the brain remains fairly unexplored with less than a handful of reports conjecturing the possible route of dissemination. Ability of *S. enterica* serovar Typhimurium 14028 in invading the human brain microvascular endothelial cells (hBMECs; *ex vivo*) has been reported. They excluded the role of previously documented *Salmonella* pathogenicity island 1 (SPI-1) locus (otherwise implicated in epithelial cell invasion) and suggested the possibility of yet another novel unexplored mechanism which might be responsible for hBMEC invasion (van Sorge et al., 2011). Another recent study, using the same *Salmonella* strain, has highlighted the role of SPI-1 and outer membrane protein A gene, aiding in host brain invasion (Chaudhuri et al., 2018). However, the exact mechanism still remains to be deciphered. Also, it was observed that the ability of bacteria to colonize one or all parts of the brain varied among individual mice as well. Therefore, the plausible role of host factors in such strain specific infections cannot be ruled out. Study is underway in our lab for elucidation of these aspects pertaining to *Salmonella* infection.

Typhoid associated neurological complications are associated with a very high mortality rate. Additional complications such as emerging antimicrobial resistance and the inability of antibiotics in treating such infections (Chaudhuri et al., 2018), suggest the use of probiotics as the most suitable recourse. Mounting evidence has highlighted the role of probiotics in influencing the gut-brain axis by modulating the gut microbiota composition (Rogers et al., 2016). At the gut interface, significant decrease in the *Salmonella* bio-burden, along with restoration of intestinal histo-architecture after *L. plantarum* (RTA 8) administration (Gill et al., 2001; De LeBlanc et al., 2010; Rodríguez-Lecompte et al., 2012; Liu et al., 2018) could be attributed to the multi-pronged ability of probiotics in enhancing competitive binding at the intestinal epithelium and reducing the luminal pH by acid production (Fayol-Messaoudi et al., 2005; De Keersmaecker et al., 2006), thus aiding in direct pathogen clearance and also preventing its subsequent systemic dissemination (Endt et al., 2010). Similar studies elucidating the ability of *Lactiplantibacillus* in preventing occurrence of *E. coli* induced meningitis by reducing its translocation across the intestinal epithelium has been reported previously (Huang et al., 2009). Significant reduction in bacterial load in other vital organs could also be attributed to these properties of *Lactiplantibacillus* strains. Interestingly, the direct gut-brain communication could have also been implicated in clearance of bacteria from the brain tissue owing to the previously documented role of vagus nerve in transmitting immune information from the gut to the brain, resulting in activation of Fos-immunoreactive cells (production of *c-Fos*, an early gene, has been used as a marker for functionally active neurons) in the hypothalamus on stimulation by *S*. Typhimurium infection (Wang et al., 2002). Furthermore, increased expression of tight junction proteins, claudin and occludin, in the *L. plantarum* (RTA 8) administered group, as revealed by immunohistochemical analysis, substantiated its role in rectifying *Salmonella* induced intestinal permeability. The observation was found to be in concordance with other studies using probiotics (Cani et al., 2009; Mennigen et al., 2009; Saulnier et al., 2013; Rokana et al., 2016) and could be ascribed to their ability in increasing IgA production, mucin expression, and prevention of cell apoptosis (Yan and Polk, 2011) along with other anti-infective mechanisms, as mentioned above.

Many gastro-intestinal conditions are associated with mental health abnormalities such as depression, anxiety, and mood disorders and occur due to disturbances in the gut-brain axis (Skonieczna-Żydecka et al., 2018). The ability of probiotics in restoring the delicate balance between these two, serves as a potential treatment alternative in the present scenario and therefore have been renamed as “psychobiotics” (Dinan et al., 2013). In the present study, administration of *L. plantarum* (RTA 8) reversed *Salmonella* induced behavioral changes such as anxiety and depressive-like symptoms, along with reduced locomotor activity. Various reports have documented the ability of probiotics in modulating the hypothalamic-pituitary-adrenal (HPA) axis by reducing the level of pro-inflammatory cytokines, thereby affecting the immune system and indirectly affecting the nervous system (Desbonnet et al., 2008, 2010). Simultaneously, direct effect of *L. plantarum* (RTA 8) on the brain neurochemistry in reversing such behavioral deficits could also be accredited to the ability of probiotics in normalization of γ-aminobutyric acid (primary inhibitory neurotransmitter in the CNS) and acetylcholine esterase, as observed in the present study. GABA deficiencies have been associated with such behavioral deficits and Alzheimer’s disease (Cryan and Kaupmann, 2005; Ramos et al., 2006; Möhler, 2012). Similarly, increase in AChE at the neuromuscular junction might have contributed to a decrease in acetylcholine concentration, thereby making it fall below the threshold potential required for nerve transmission, resulting in cognitive decline and muscle fatigue. Since AChE is also considered to be an initiator in oxidative stress and plays a role in memory dysfunction, learning/memory loss associated with *Salmonella* infection of the brain, as documented by Chaudhuri et al. (2018), could also be due to this facet. The effect of these molecules might be mediated *via* the vagal nerve activation or modulation of the enteric nervous system by them (Bercik et al., 2011; Bravo et al., 2011). Anti-depressant effects of *Lactobacilli* and *Bifidobacteria* in various mouse models and their ability to provide relief in various stressful conditions have been reported previously which further lends weight to our findings (Savignac et al., 2014; Liu et al., 2016; Dhaliwal et al., 2018).

Probiotic administration has been reported to be effective in ameliorating infection induced behavioral abnormalities in case of *Trichuris muris* and *Citrobacter rodentium* although the information remains sparse in case of other pathogens (Lyte et al., 2006; Bercik et al., 2010; Gareau et al., 2011). To the best of our knowledge, the present study is the first report, wherein a potential probiotic strain has been used to prevent typhoid associated neurological manifestations. Previously, elaborative studies on the pathogenesis of enteric fever have revealed higher incidence of bacteremia in the first week post infection and the count has been reported to decrease thereafter with increase in duration of illness (Wain et al., 1998), eventually resulting in other pyogenic infections including osteomyelitis, pericarditits, etc. (Huang and DuPont, 2005). Considering the 14-day incubation period of *S. typhi* to cause typhoid in humans, administration of probiotic even within a period of 7-day post infection (wherein blood cultures are found to be positive in 90% cases and decline thereafter), may at least, prevent the host from developing these complications.

Since there is a dearth of studies on infection induced psychological stress and the ability of probiotic in influencing the same *via* modulation of gut-brain axis, the study provides important observations that might prove useful in devising strategies to prevent occurrence of critical illness. Further, as higher incidence of typhoid related neurological complications have been more commonly reported in infants, probiotic administration presents as a safe and efficacious option which further adds to the translational robustness of this study.

## Data Availability Statement

All datasets presented in this study are included in the article/supplementary material.

## Ethics Statement

The animal study was reviewed and approved by Institutional Animal Ethics Committee, Panjab University, Chandigarh, India (Approval No. PU/45/99/CPCSEA/IAEC/2018/225).

## Author Contributions

PR conceived and designed the study. AK, PR, IK, and KC were involved in formal analysis. Investigations were carried out by AK. Methodological assistance was provided by KC and PR. Project administration was done by PR, KC, and IK. Resources were provided by KC and PR. The study was supervised by PR, KC, and IK. Original draft preparation was done by AK and PR. Writing, reviewing, and editing in the manuscript were done by AK, PR, and IK. All authors contributed to the article and approved the submitted version.

### Conflict of Interest

The authors declare that the research was conducted in the absence of any commercial or financial relationships that could be construed as a potential conflict of interest.
